# Naturally Acquired Genotype‐Specific HPV Seroreactivity and Subsequent HPV Detection Among Heterosexual Partners

**DOI:** 10.1002/jmv.70163

**Published:** 2025-01-15

**Authors:** Kristy Ng, Mariam El‐Zein, Michel D. Wissing, Ann N. Burchell, Pierre‐Paul Tellier, François Coutlée, Tim Waterboer, Eduardo L. Franco

**Affiliations:** ^1^ Division of Cancer Epidemiology McGill University Montreal Quebec Canada; ^2^ MAP Centre for Urban Health Solutions, Li Ka Shing Knowledge Institute, St. Michael's Hospital, Unity Health Toronto Toronto Ontario Canada; ^3^ Department of Family and Community Medicine Temerty Faculty of Medicine, University of Toronto Toronto Ontario Canada; ^4^ Department of Family Medicine McGill University Montreal Quebec Canada; ^5^ Laboratoire de Virologie Moléculaire, Centre de recherche du Centre Hospitalier de l'Université de Montréal Montreal Quebec Canada; ^6^ Départements de Microbiologie, Infectiologie et Immunologie, et de Gynécologie‐Obstétrique Université de Montréal Montreal Quebec Canada; ^7^ Départements de Médecine, de Médecine clinique de Laboratoire et d'Obstétrique‐Gynécologie Centre Hospitalier de l'Université de Montréal Montreal Quebec Canada; ^8^ Infections and Cancer Epidemiology Division German Cancer Research Center Heidelberg Germany

**Keywords:** human papillomavirus, natural immunity, serology, transmission

## Abstract

The protective effect of naturally acquired humoral immunity against human papillomavirus (HPV) infection remains unclear. To investigate the role of infection‐induced antibodies on HPV detection in heterosexual partners, we used data from 392 unvaccinated couples (females aged 18–25 years attended up to six visits over 2 years; males aged 17–37 years attended up to two visits 4 months apart) enrolled (2005–2011) in Montreal. Genital and blood samples were HPV DNA genotyped and tested for L1 antibody titers of 14 HPV genotypes. Analyses considered female‐HPV units (*n* = 4914 based on 351 couples) and male‐HPV units (*n* = 4214 based on 301 couples); each female and male, respectively, contributed up to 14 observations corresponding to 14 genotypes. Modified Cox and logistic regressions estimated hazard and odds ratios (HR/OR) and 95% confidence intervals (CIs) for genotype‐specific HPV detections by partner serostatus (high/low: ≥/< baseline median antibody titers, 392 couples). There were 919 and 231 cumulative HPV detections among female‐HPV and male‐HPV units, respectively. Risk of HPV detections in females (HR = 1.05, CI: 0.90–1.22) and males (OR = 1.31, CI: 0.97–1.77) was similar between those with partners of high versus low serostatus. Constraining to baseline HPV‐negative participants with HPV‐positive partners yielded unchanged results. This lack of association suggests that naturally developed HPV antibodies do not protect sexual partners from infection.

## 1 Introduction

Human papillomavirus (HPV) is among the most common sexually transmitted infections worldwide [1]. While up to 90% of HPV infections clear within 2 years, persistent infection with high‐risk HPV genotypes (i.e., HPVs 16 and 18) is a necessary cause of cervical cancer and an important cause of other anogenital and head‐and‐neck cancers [2]. Studies on prophylactic HPV vaccination demonstrated its ability to provide strong, long‐term protection against vaccine‐targeted HPV‐genotype infections and related genital diseases for vaccinated individuals [3, 4]. Furthermore, a previous study investigating HPV transmission dynamics in couples reported that, among sexually active females who were vaccinated before study enrollment, transmission of vaccine‐targeted HPV genotypes to their male partners was reduced relative to couples with unvaccinated females [5]. Since virtually all (> 90%) HPV‐vaccinated young adults seroconvert and develop high peak antibody titers postvaccination [6, 7], serum antibodies likely play an important role in the control and transmission of HPV infections by preventing host cell entry [8, 9].

Natural HPV infections can likewise induce humoral immune responses; however, seroconversion rates following genital infection with high‐risk HPV are in the range of 40%–60% in females and 4%–36% in males [10, 11, 12]. Among those who seroconvert, antibody titers are often much lower than the levels induced by vaccination [12]. Research on the effect of naturally developed antibodies on subsequent HPV detection at the individual level is inconclusive; several studies have reported that such antibodies have no effect in both females and males [13, 14, 15, 16, 17, 18, 19], while a recent meta‐analysis found they provide partial protection for genital HPV16 among females [20]. Nevertheless, given that the level of protection provided by naturally developed serum antibodies is suggested to be much lower than the one induced by vaccination, it is unclear whether protection conferred by naturally developed antibodies extends beyond the individual, that is, does it help prevent infection in their sexual partners?

The objective of this study was to examine the effect of infection‐induced naturally developed serum antibodies on HPV transmission dynamics between sexual partners. Specifically, we assessed the risk of subsequent cumulative and incident genital HPV detection in an individual with respect to the partner's baseline serostatus using survival models for multiple failure‐time data at the HPV level. We hypothesized that naturally developed antibodies may offer some level of protection to sexual partners, though likely not a robust one. Analyses were based on a population in which HPV transmission most often likely occurs: a cohort of young adults in recently formed sexual relationships from the HPV Infection and Transmission among Couples through Heterosexual activity (HITCH) study [21]. Given most HPV infections are transient, the HITCH study was uniquely designed to specifically target these newly forming partnerships and longitudinally investigate HPV infection and transmission patterns at the couple level.

## 2 Methods

### 2.1 Study Population

Details of the HITCH cohort study were described previously [21]. We enrolled 502 heterosexual couples (an assigned male at birth and an assigned female at birth) between 2005 and 2011 in Montreal, Canada, consisting of young adult females (aged 18–24 years) attending a postsecondary institution and their male partners (≥ 18 years). This population of young adult females was targeted since rates of HPV infection are highest in this age group [22]. Couples were eligible if they began their sexual relationship within 6 months before enrollment as a way to maximize the frequency of true detections of HPV transmission. The female had to be neither pregnant nor planning to become pregnant in the following 2 years, had an intact uterus, and had no history of cervical lesions or cancer. Females attended up to six study visits over the 2‐year follow‐up period (i.e., 0, 4, 8, 12, 18, and 24 months). Males attended up to two study visits (i.e., 0 and 4 months). At each visit, participants provided, (i) nurse‐collected blood samples and (ii) self‐collected vaginal samples (females) or nurse‐collected penile and/or scrotal samples (males). Couples were asked to refrain from sexual activity for 24 h before sample collection to minimize genital sample contamination. In addition, participants completed self‐administered web‐based questionnaires at baseline and over follow‐up, which captured repeated measurements of demographics, sexual behavior and history, and HPV vaccination. In the current analysis, we focused exclusively on unvaccinated couples.

The HITCH study abides by national and international guidelines regarding research with human data and materials, including the Declaration of Helsinki. The study was conducted in accordance with the principles and articles specified by the Tri‐Council Policy Statement Ethical Conduct for Research Involving Humans.

### 2.2 HPV Genotyping and Multiplex Serology

Genital samples were tested for the presence of HPV DNA from 36 HPV genotypes by polymerase chain reaction (PCR), based on the amplification of a 450 base‐pair sequence of the L1 capsid gene using the Linear Array HPV genotyping assay (Roche Molecular Systems, Laval, Canada) [23]. A β‐globin DNA sequence was co‐amplified as a control for the presence of cells, DNA integrity, and the absence of inhibitors; a sample was considered valid if β‐globin DNA was detected.

Sera from blood samples were tested for reactivity against antibodies targeting the major capsid protein (L1) of 12 high‐risk carcinogenic and probably carcinogenic HPV genotypes (16, 18, 31, 33, 35, 39, 45, 51, 52, 58, 59, and 68) [2] and two HPV genotypes (6, 11) that most commonly cause genital warts, using a glutathione *S*‐transferase (GST) fusion protein‐based multiplex serology assay at the German Cancer Research Center (DKFZ) in Heidelberg, Germany [24]. In brief, HPV L1 proteins were expressed in *Escherichia coli* as GST‐L1‐tag fusion proteins and loaded onto sets of glutathione‐derivatized, spectrally distinct polystyrene beads (Luminex). Sera were preincubated with polyvinyl alcohol, polyvinylpyrrolidone, and Super ChemiBlock (Chemicon) to block nonspecific binding of antibodies to beads, and then incubated with the bead sets. Bound antibodies were detected with a triple‐specific biotinylated anti‐human immunoglobulin A (IgA), IgM and IgG secondary antibody, and streptavidin‐R‐phycoerythrin. Primary serum antibodies were quantified with a Luminex 200 flow cytometer as median fluorescence intensity (MFI). Hereafter, HPV GST‐L1 antibodies are referred to simply as genotype‐specific HPV antibodies.

### 2.3 Analytical Frameworks

All analyses were conducted at the genotype‐specific HPV level, that is, a participant was followed throughout the study for HPV DNA positivity for each of the 14 tested HPVs. The combination of a unique participant and HPV genotype was considered as a participant‐HPV unit; therefore, each individual contributed 14 participant‐HPV units. The baseline serostatus of the participant's partner was also genotype‐specific, corresponding to the participant‐HPV unit of the same HPV genotype.

**Figure 1:**
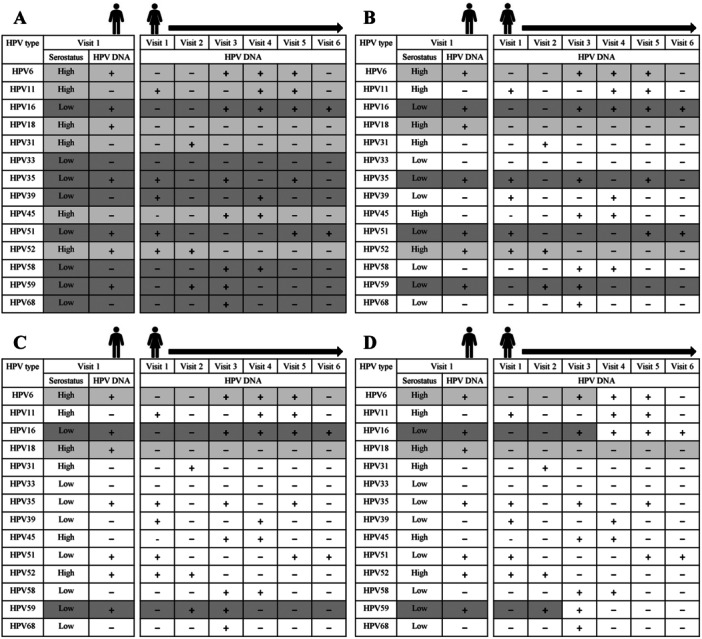
Analytical frameworks to assess the association between male serostatus and female HPV detection. Illustrated is a fictitious partnership applied to four genotype‐specific analytical frameworks, which consider couples who were unvaccinated and provided baseline genital samples. Here, the male partner provided a blood sample at baseline and the female had HPV DNA data at follow‐up. In each panel, the left columns indicate the male serostatus and genotype‐specific HPV DNA positivity (“+”) at baseline for each of the 14 tested HPV genotypes, while the right columns follow the female partner's HPV DNA positivity to the end of the study/censorship. Each row consisting of a unique HPV genotype is considered a female‐HPV unit. Shaded cells indicate the time contributed to the analysis and the exposure of male genotype‐specific serostatus (light gray: high serostatus, dark gray: low serostatus). (A) Least constrained framework: cumulative HPV detections among all female‐HPV units. (B) Less constrained framework: cumulative HPV detections among female‐HPV units with HPV‐positive males at baseline. (C) Moderately constrained framework: cumulative HPV detections among female‐HPV units who initially tested HPV‐negative at baseline and are susceptible to acquiring an infection from an HPV‐positive male. (D) Most constrained framework: incident HPV detections among female‐HPV units who initially tested HPV‐negative at baseline and are susceptible to acquiring an infection from an HPV‐positive male. DNA, deoxyribonucleic acid; HPV, human papillomavirus.

**Figure 2:**
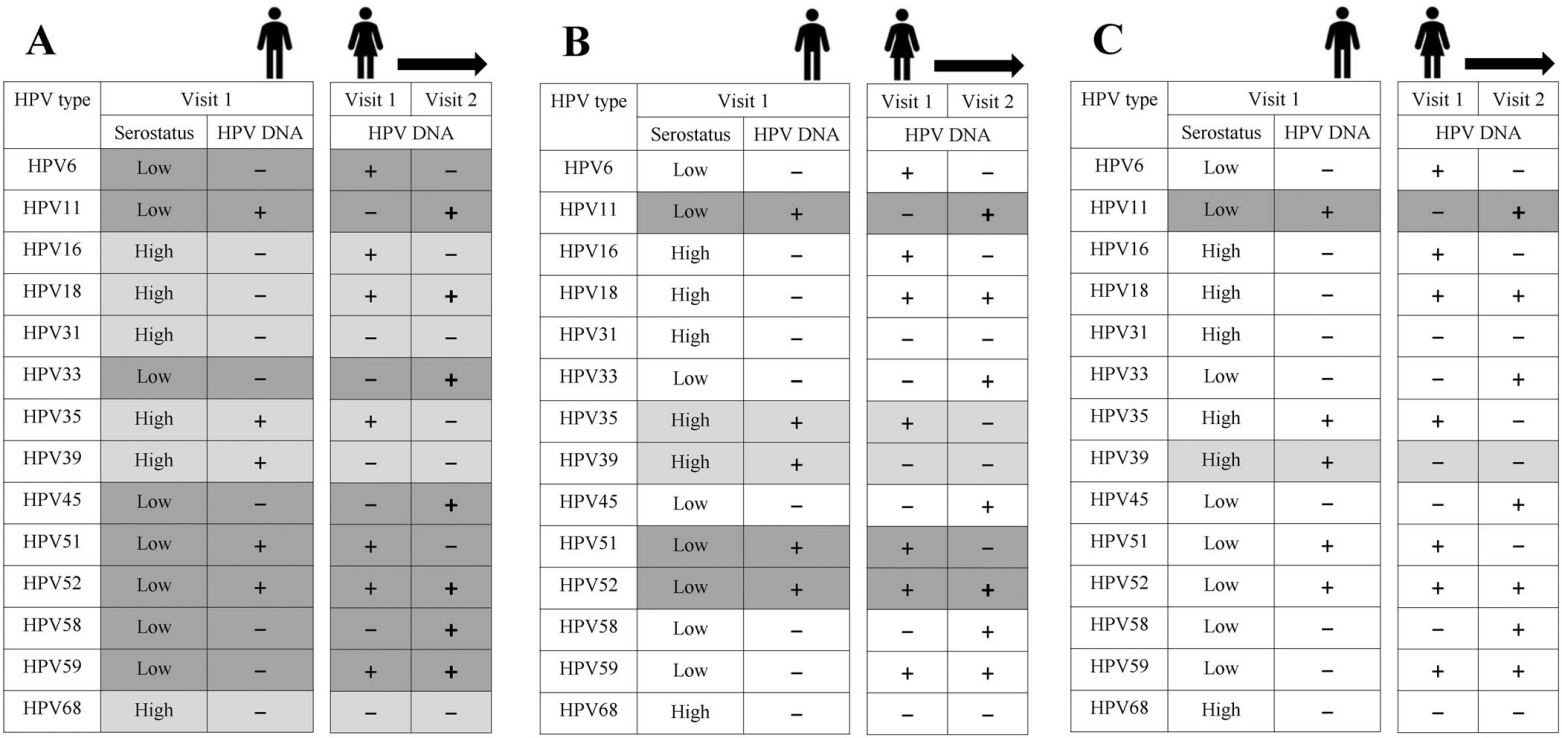
Analytical frameworks to assess the association between female serostatus and male HPV detection. Illustrated is a fictitious partnership applied to three genotype‐specific analytical frameworks, which consider couples who were unvaccinated and provided baseline genital samples. Here, the female partner provided a blood sample at baseline and the male had HPV DNA data at follow‐up. In each panel, the left columns indicate the female serostatus and genotype‐specific HPV DNA positivity (“+”) at baseline for each of the 14 tested HPV genotypes, while the right columns follow the male partner's HPV DNA positivity to the end of the study/censorship (Visit 2). Each row consisting of a unique HPV genotype is considered a male‐HPV unit. Shaded cells indicate the time contributed to the analysis and the exposure of female genotype‐specific serostatus (light gray: high serostatus, dark gray: low serostatus). (A) Least constrained framework: HPV detections among all male‐HPV units. (B) Less constrained framework: HPV detections among male‐HPV units with HPV‐positive females at baseline. (C) Most constrained framework: HPV detections among male‐HPV units who initially tested HPV‐negative at baseline and are susceptible to acquiring an infection from an HPV‐positive female. DNA, deoxyribonucleic acid; HPV, human papillomavirus.

To assess the association between a partner's baseline genotype‐specific HPV serostatus and subsequent corresponding genital HPV detection in the participant, we devised four analytical frameworks, each with increasing constraints (from least to most) on (i) the inclusion of participant‐HPV units based on the couples’ baseline genotype‐specific HPV detections, and (ii) the definition of HPV detection (cumulative or incident detection). For each participant‐HPV unit, cumulative detections included all HPV‐positive events throughout follow‐up, while incident detections only included the first occurrence of an HPV detection at follow‐up. Figure 1 is a schematic representation of the analytical frameworks to specifically assess the association between a male partner's baseline genotype‐specific HPV serostatus and subsequent vaginal HPV detection of the female. These included the (1) least constrained: cumulative HPV detections among all female‐HPV units irrespective of the couple's baseline HPV DNA detection status (i.e., most liberal analytical approach); (2) less constrained: cumulative HPV detections among female‐HPV units with HPV‐positive male partners at baseline; (3) moderately constrained: cumulative HPV detections among HPV‐negative female‐HPV units susceptible to acquiring an HPV infection from an HPV‐positive male at baseline; and (4) most constrained: incident HPV detections among HPV‐negative female‐HPV units susceptible to acquiring an HPV infection from an HPV‐positive male at baseline (i.e., most conservative analytical approach). Similarly, Figure 2 illustrates three analytical frameworks (given that there was a single follow‐up visit for male HPV detection) of increasing constraints on the inclusion of male‐HPV units. These included (1) least constrained: HPV detections among all male‐HPV units; (2) less constrained: HPV detections among male‐HPV units with HPV‐positive females at baseline; and (3) most constrained: HPV detections among HPV‐negative male‐HPV units susceptible to acquiring an HPV infection from an HPV‐positive female at baseline.

### 2.4 Statistical Analyses

Participants who had invalid or missing genital samples at baseline were assigned the HPV DNA positivity results of their first follow‐up visit (males: *n* = 8; females: *n* = 4). A male and a female were considered genotype‐specific HPV DNA positive at the time of the study visit if the penile or scrotal sample and the vaginal sample, respectively, were positive for a given HPV genotype. To interpret seroreactivity, a male baseline genotype‐specific HPV serostatus was defined as low or high using the genotype‐specific median baseline antibody titer of all 392 unvaccinated males with serological data as the cut‐off. Likewise, a female's baseline genotype‐specific HPV serostatus was defined as low or high using the female (*n* = 391) genotype‐specific median baseline antibody titer as the cut‐off. Since the median time to seroconversion is generally 12+ months following an incident genital HPV infection [10, 25], three males and eleven females with missing serological data at baseline were assigned the values of serological titers obtained at their first follow‐up visit, which by design occurred 4 months post‐enrollment.

Descriptive statistics were used to summarize participants' characteristics at baseline, separately for males and females. Based on the above‐mentioned analytical frameworks, hazard ratios (HRs) and 95% confidence intervals (CIs) were estimated for cumulative and incident vaginal HPV detections among females with respect to the male partner baseline serostatus using the Prentice, Williams, and Peterson‐total time (PWP‐TT) model, an extension of the Cox proportional hazard model. The PWP‐TT model is used for multiple failure‐time data and considers cumulative HPV DNA detections as ordered recurrent events, assuming that the occurrence of the first genotype‐specific HPV detection increases the likelihood of recurrence [26]. The Efron method handled any tied events [27, 28, 29]. Considering only the most constrained analytical framework, we estimated Kaplan‐Meier failure curves stratified by male partner baseline serostatus. Binary logistic regression was used to determine odds ratios (ORs) and 95% CIs of penile/scrotal HPV detection at the second study visit among males with respect to the female partner baseline serostatus. All CIs accounted for repeated measurements of multiple HPV genotypes per individual; observations were clustered by participant.

In addition, we calculated, separately for males and females using forward stepwise logistic regression, the propensity for HPV infection at baseline based on sexual behavior characteristics, according to each individual reporting within couples. Using the Wald test method, we chose a *p* value < 0.2 to retain all the independent variables that were correlated with the risk of an HPV infection. Candidate variables included marital status, lifetime number of sex partners, lifetime number of vaginal sex partners, time since first sexual activity, time since first vaginal sexual intercourse, weekly frequency of sexual activity, weekly frequency of vaginal sexual activity, frequency of condom use, and whether the individual had concurrent partners. For numeric variables, missing values were imputed based on the mean. We used the regression coefficients of the retained variables to construct a linear propensity score and repeated the above‐mentioned analyses (i.e., female HPV detection and male HPV detection by partner baseline serostatus) restricting to participants who were categorized within the highest tertile of the propensity score. We refer to these as restricted analyses.

Two sensitivity analyses were conducted. First, we excluded couples with no baseline genital samples and repeated the analyses. Secondly, we reassessed the association between male partner serostatus and female HPV detection, truncating the number of recurrent HPV detections to a maximum of three, as estimates from PWP‐TT models may become unstable due to smaller risk sets in later HPV detection events [26].

Statistical analyses were performed using Stata 18.0 (StataCorp LLC, College Station, Texas).

## 3 Results

As shown in Table 1, the median age of females and males were 21 (standard deviation [SD] = 1.76) and 22 (SD = 3.47), respectively. Most were White (females: 81.19%; males: 85.35%), and 62.05% and 51.03% of females and males, respectively, had never smoked. The median age at first vaginal intercourse was 17 years for both sexes. The median number of lifetime vaginal sex partners for participants was 5 (female interquartile range [IQR] = 2–8, male IQR = 3–11), and weekly frequency of sexual activity (oral, vaginal, and/or anal) for females and males was 5 (IQR = 3.00–6.00) and 4 (IQR = 3.00‐6.19), respectively. The majority of participants noted they rarely used condoms (females: 30.60%, males: 30.22%), and did not have additional partners to their HITCH counterpart (females: 76.15%, males: 86.41%). Of the 14 examined HPV genotypes, HPV16 was the most prevalent at baseline (females: 19.74%, males: 17.99%), followed by HPV51 (females: 10.26%, males: 12.34%).

**Table 1:** Baseline characteristics of unvaccinated participants with valid blood and genital samples in the HITCH cohort study.

Variable	Females (*n* = 390)	Males (*n* = 390)
Age,^a^ mean (SD)	21 (1.76)	22 (3.47)
Ethnicity, *n* (%)		
White	315 (81.19)	332 (85.35)
Asian	33 (8.51)	14 (3.60)
Latino	18 (4.64)	22 (5.66)
Black	12 (3.09)	15 (3.86)
Mixed	10 (2.58)	6 (1.54)
*Missing*	2	1
Smoking, *n* (%)		
Never	242 (62.05)	199 (51.03)
Ever (current/former)	148 (37.95)	191 (48.97)
Age at first vaginal intercourse, median (IQR)	17 (15‐18)	17 (16‐18)
*Missing*	3	3
Number of lifetime vaginal sex partners,^b^ *n* (%)		
Median (IQR)	5 (2.00–8.00)	5 (3.00–11.00)
1–5	219 (56.74)	196 (50.91)
6–10	98 (25.39)	86 (22.34)
11–15	47 (12.18)	52 (13.51)
≥ 16	22 (5.70)	51 (13.25)
*Missing*	4	5
Weekly frequency of sexual activity,^c^ *n* (%)		
Median (IQR)	5 (3.00–6.00)	4 (3.0–6.19)
≤ 5	237 (65.65)	254 (65.46)
> 5–10	102 (28.25)	108 (27.84)
> 10–15	10 (2.77)	21 (5.41)
> 15	12 (3.32)	5 (1.29)
*Missing*	29	2
Condom use,^d^ *n* (%)		
Never (0%)	48 (13.11)	47 (12.91)
Rarely (1%–25%)	112 (30.60)	110 (30.22)
Some of the time (26%–75%)	58 (15.85)	57 (15.66)
Most of the time (76%–99%)	73 (19.95)	73 (20.05)
Always (100%)	75 (20.49)	77 (21.15)
*Missing*	24	26
Have concurrent partner(s), *n* (%)		
Yes	93 (23.85)	53 (13.59)
No	297 (76.15)	337 (86.41)
Genotype‐specific HPV prevalence^e^, *n* (%)		
HPV6	17 (4.36)	24 (6.17)
HPV11	3 (0.77)	2 (0.51)
HPV16	77 (19.74)	70 (17.99)
HPV18	13 (3.33)	13 (3.34)
HPV31	20 (5.13)	16 (4.11)
HPV33	5 (1.28)	2 (0.51)
HPV35	4 (1.03)	4 (1.03)
HPV39	28 (7.18)	31 (7.97)
HPV45	7 (1.79)	6 (1.54)
HPV51	40 (10.26)	48 (12.34)
HPV52	31 (7.95)	21 (5.40)
HPV58	19 (4.87)	12 (3.08)
HPV59	23 (5.90)	22 (5.66)
HPV68	13 (3.33)	9 (2.31)

Abbreviations: IQR, interquartile range; SD, standard deviation.

^a^ Age range was 18–25 years among females (only one female was 25 years at enrollment, her male partner was 23 years of age) and 17–37 among males (only one male was 17 years old at enrollment, his female partner was 18 years of age).

^b^ Number of lifetime vaginal sex partners of the other sex.

^c^ Weekly frequency of sexual activity reported by the study participant, including oral, vaginal, and/or anal sex.

^d^ Frequency of condom use for vaginal sex with the HITCH partner, according to the study participant.

^e^ Genotype‐specific HPV prevalence was determined with genital samples (vaginal or penile and scrotal). If both were available, male penile and scrotal samples were merged. One male lacked baseline and follow‐up genital samples (*n* = 389). Refer to Figure 3.

Supporting Information S1: Tables S1 and S2 present the sexual behavior variables and corresponding coefficients used in the forward stepwise logistic regressions to estimate the propensity of HPV positivity in each of females (*n* = 390) and males (*n* = 389) with available genital samples at baseline. Categorizing the resulting score into tertiles of least to highest propensity, the corresponding baseline prevalence of any HPV positivity in females was 14.62%, 43.85%, and 70.00%. Equivalent values for males were 12.21%, 41.86%, and 73.64%.

**Figure 3:**
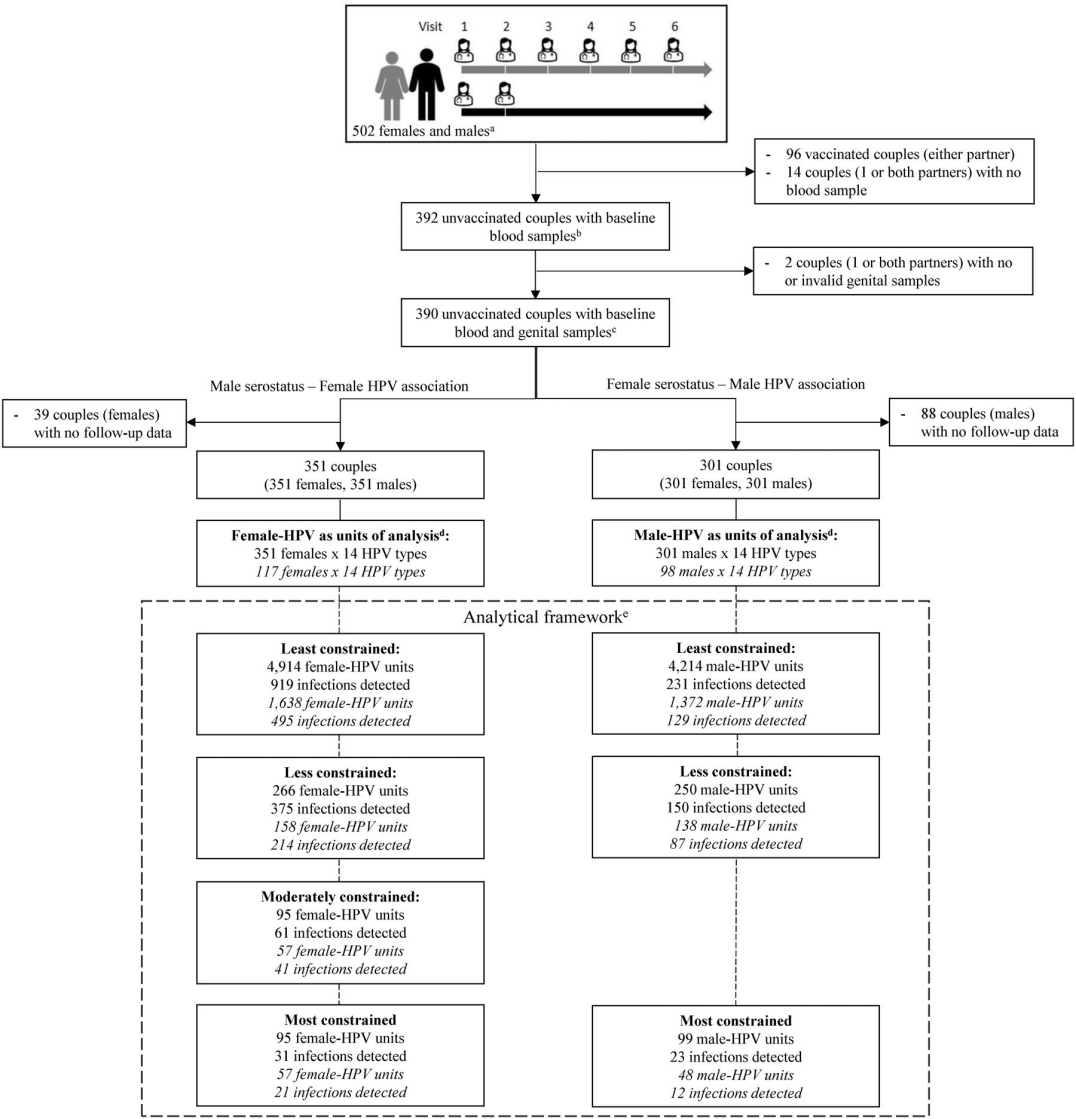
Analysis sample and participant‐HPV infections detected in the HITCH study. → Indicates changes in observational sample size. — Indicates data transformation from observational to analysis sample units. ‐ ‐ ‐ ‐ ‐ Indicates breakdown of analysis sample. Regular text: Indicates analysis sample and HPV detections included in unrestricted analyses. *Italicized* text: Indicates analysis sample and HPV detections included in restricted analyses. Refer to Section 2 and Supporting Information S1: Tables S1 and S2. ^a^Includes HITCH females and their first enrolled male partners. ^b^Eleven females and three males (from 14 couples) with no baseline serology were assigned serology results of those obtained at the first follow‐up visit. One additional female had neither serology data at baseline nor at the first follow‐up visit; the couple was removed from the female serostatus—male HPV association analysis. ^c^Four females and eight males (from 12 couples) with no baseline genital samples were assigned genital HPV DNA results of those obtained at the first follow‐up visit. One additional male lacked baseline and follow‐up genital samples, but his female partner had genital samples at baseline and follow‐up, qualifying the couple for the least constrained analytical framework in the male serostatus‐female HPV association analysis. ^d^Each female from a couple can have detectable HPV DNA of any of the 14 unique HPV genotypes, thus contributing up to 14 observations. This combination of a unique female and HPV genotype is considered a “female‐HPV unit” at risk for cumulative or incident HPV detection until the end of the study, up to five follow‐up visits. Male‐HPV units have only one follow‐up visit. ^e^Least constrained includes cumulative HPV detections among all participant‐HPV units, less constrained includes cumulative HPV detections among participant‐HPV units with HPV‐positive partners at baseline, moderately constrained includes cumulative HPV detections among HPV‐negative participant‐HPV units susceptible to acquiring an infection from an HPV‐positive partner at baseline, and most constrained includes incident HPV detections among HPV‐negative participant‐HPV units susceptible to acquiring an infection from an HPV‐positive partner at baseline. Refer to Figures 1 and 2.

Figure 3 details the analysis samples and count of HPV infections by analytical framework and analysis (unrestricted and restricted). For the assessment of the association between male partner baseline serostatus and female genotype‐specific HPV detection, 351 couples were included. In the unrestricted least, less, and moderately constrained analytical frameworks, there were 919 cumulative HPV detections in 4914 female‐HPV units, 375 in 266 female‐HPV units, and 61 in 95 female‐HPV units, respectively. In the most constrained analytical framework, 31 incident HPV detections were found in 95 female‐HPV units. For the assessment of the association between female partner baseline serostatus and male genotype‐specific HPV detection (301 couples), there were 231 HPV detections in 4214 male‐HPV units, 150 in 250 male‐HPV units, and 23 in 99 male‐HPV units, in the least, less, and most constrained analytical frameworks, respectively. Evidently, the number of HPV detections and participant‐HPV units were fewer in the restricted analyses, as shown in Figure 3.

Table 2 presents the findings of unrestricted and restricted analyses for the association between male partner baseline serostatus and female genotype‐specific HPV detection. Among all female‐HPV units (least constrained) and female‐HPV units with partners that tested HPV positive (less constrained), the HRs of cumulative HPV detections of those with a partner of high baseline serostatus relative to low were 1.05 (CI: 0.90–1.22) and 0.98 (CI: 0.78–1.24), respectively; the differences in hazard were not statistically significant in these analytical frameworks. The HRs of cumulative and incident HPV detection were the greatest in magnitude above the null in women “at risk” of HPV infection, where the female‐HPV unit was HPV‐negative but her male‐partner was HPV‐positive at baseline (moderately constrained: HR = 1.60, CI: 0.94–2.73; most constrained: HR = 1.15, CI: 0.56‐2.35), though results remained nonsignificant and with larger CIs. Similar findings were observed considering the restricted female study population; corresponding HRs from least to most constrained analytical frameworks were 1.14 (CI: 0.94–1.37), 1.05 (CI: 0.81–1.37), 1.47 (CI: 0.73–2.95); and 1.21 (CI: 0.49–2.94). The hazard of HPV detection across the four analytical frameworks remained non‐significantly greater in females with high partner baseline serostatus relative to low.

**Table 2:** Hazard ratios of female genotype‐specific HPV detections according to male serostatus defined by median antibody titer. Results are presented by analytical framework and by applied restrictions on the propensity of HPV infection.

Analytical framework^a^	Male genotype‐specific serostatus^b^	Unrestricted analysis				Restricted analysis^e^			
		No. of events^c^	No. of female‐HPV units	No. of months contributed by female‐HPV units	Hazard ratio^d^ (95% CI)	No. of events^c^	No. of female‐HPV units	No. of months contributed by female‐HPV units	Hazard ratio^d^ (95% CI)
Least constrained	Low	430	2357	59 258.44	1 [Reference]	253	802	19 107.67	1 [Reference]
	High	489	2557	63 320.33	1.05 (0.90, 1.22)	242	836	18 531.39	1.14 (0.94, 1.37)
Less constrained	Low	187	130	3114.79	1 [Reference]	116	87	2050.30	1 [Reference]
	High	188	136	3147.75	0.98 (0.78, 1.24)	98	71	1668.69	1.05 (0.81, 1.37)
Moderately constrained	Low	21	43	1095.28	1 [Reference]	14	27	673.19	1 [Reference]
	High	40	52	1205.41	1.60 (0.94, 2.73)	27	30	727.11	1.47 (0.73, 2.95)
Most constrained	Low	14	43	913.66	1 [Reference]	10	27	557.74	1 [Reference]
	High	17	52	938.66	1.15 (0.56, 2.35)	11	30	523.34	1.21 (0.49, 2.94)

Abbreviations: CI, confidence interval; HPV, human papillomavirus.

^a^ Least constrained includes cumulative HPV detections among all female‐HPV units, less constrained includes cumulative HPV detections among female‐HPV units with baseline HPV‐positive males, moderately constrained includes cumulative HPV detections among HPV‐negative female‐HPV units susceptible to acquiring an infection from an HPV‐positive male at baseline, and most constrained includes incident HPV detections among HPV‐negative female‐HPV units susceptible to acquiring an infection from an HPV‐positive male at baseline. Refer to Figure 1.

^b^ Male genotype‐specific serostatus for each female‐HPV unit was defined as low or high using the male median baseline antibody titer for the corresponding HPV genotype as the cut‐off.

^c^ Indicates the count of cumulative or incident HPV detections (i.e., sum of events across all HPV genotypes), dependent on the analytical framework. Detectable HPV DNA include each of the 14 unique HPV genotypes that could be present in each female from a partnership; a female can contribute multiple events based on her genotype‐specific HPV detections.

^d^ Hazard ratios were determined using the Prentice, Williams, and Peterson‐total time model for multiple failure‐time survival data.

^e^ Hazard ratios were restricted to the highest tertile of the female's propensity score calculated at the female level using a forward stepwise logistic regression model: dependent variable = any HPV positivity at baseline; independent variables = couple sexual behavior characteristics at baseline. Refer to Section 2 and Supporting Information S1: Table S1.

The Kaplan–Meier curves for the most constrained analytical framework further showed the lack of significant difference in the risk of incident HPV detection among all female‐HPV units with a male partner of high baseline serostatus versus low (Supporting Information S1: Figure S1).

The change in odds of male genotype‐specific HPV detection according to female partner baseline serostatus was also not statistically significant (Table 3). In the least, less, and most constrained analytical frameworks of the unrestricted male population, corresponding ORs were 1.31 (CI: 0.97–1.77), 0.99 (CI: 0.58–1.67), and 0.76 (CI: 0.25–2.33), suggesting an inconclusive increase in the odds of genital HPV detection in those with high female partner baseline serostatus relative to low among all male‐HPV units (least constrained), but an inconclusive decrease among men “at risk” of HPV infection (most constrained). Nonsignificant associations were also observed in the restricted analyses for males; corresponding ORs were 1.26 (CI: 0.83–1.92), 1.11 (CI: 0.53–2.32), and 0.25 (CI: 0.06–1.11).

**Table 3:** Odds ratios of male genotype‐specific HPV detections according to female serostatus defined by median antibody titer. Results are presented by analytical framework and by applied restrictions on the propensity of HPV infection.

Analytical framework^a^	Female genotype‐specific serostatus^b^	Unrestricted analysis			Restricted analysis^d^		
		Male‐HPV detection^c^		Odds Ratio (95% CI)	Male‐HPV detection^c^		Odds Ratio (95% CI)
		Positive	Negative		Positive	Negative	
Least constrained	Low	96	1924	1 [Reference]	50	552	1 [Reference]
	High	135	2059	1.31 (0.97, 1.77)	79	691	1.26 (0.83, 1.92)
Less constrained	Low	53	35	1 [Reference]	32	20	1 [Reference]
	High	97	65	0.99 (0.58, 1.67)	55	31	1.11 (0.53, 2.32)
Most constrained	Low	9	25	1 [Reference]	8	12	1 [Reference]
	High	14	51	0.76 (0.25, 2.33)	4	24	0.25 (0.06, 1.11)

Abbreviations: CI, confidence interval; HPV, human papillomavirus.

^a^ Least constrained includes HPV detections among all male‐HPV units at follow‐up (Visit 2), less constrained includes HPV detections among male‐HPV units with baseline HPV‐positive females, and most constrained includes HPV detections among HPV‐negative male‐HPV units susceptible to acquiring an infection from an HPV‐positive female at baseline. Refer to Figure 2.

^b^ Female genotype‐specific serostatus for each male‐HPV unit was defined as low or high using the female median baseline antibody titer for the corresponding HPV genotype as the cut‐off.

^c^ Indicates the cumulative number of HPV‐male units that are HPV‐positive or HPV‐negative at follow‐up. Detectable HPV infections include each of the 14 unique HPV genotypes that could be present in each male from a partnership; a male can contribute multiple events based on his genotype‐specific HPV detections.

^d^ Odds ratios were restricted to the highest tertile of the male's propensity score calculated at the male level using a forward stepwise logistic regression model: dependent variable = any HPV positivity at baseline; independent variables = couple sexual behavior characteristics at baseline. Refer to Section 2 and Supporting Information S1: Table S2.

Conclusions remained virtually identical when couples with no baseline genital samples were excluded from the analyses (results not shown). HRs of all analytical frameworks for female HPV detection considering male partner baseline serostatus remained statistically insignificant when the number of recurrent HPV detections was truncated.

## 4 Discussion

We found no evidence of change in subsequent genital HPV DNA detection of the 14 tested genotypes considering naturally acquired partner serostatus in a cohort of young, recently formed, sexually active, heterosexual couples. We showed that this lack of association was seen in both females and males and their respective partners’ baseline serostatus of the opposite sex. Our findings suggest that naturally developed HPV antibodies may not confer significant protection among sexual partners.

To our knowledge, this is the first study directly exploring the effects of one's serostatus as a possible influence on the transmission of the same HPV genotype to heterosexual partners. Female partners of males with high baseline serostatus did not have appreciably different risks of cumulative or incident HPV detection relative to those with partners of low serostatus. Interestingly, albeit nonsignificant, the HRs for cumulative and incident HPV detection in females “at risk” of HPV infection (moderately and most constrained analytical frameworks) estimated a greater relative risk of vaginal HPV detection among females with high male partner baseline serostatus, even after restricting to those of increased propensity for HPV infection by sexual behavior characteristics. Similarly, previous studies at the individual level (i.e., not couple‐based) found no evidence of protective effects of males' baseline HPV seropositivity against subsequent penile/scrotal HPV infection (specifically HPVs 6, 11, 16, and 18) [16, 17, 18, 19]. Conversely, a recent meta‐analysis noted that the risk of incident genital HPV16 detection among baseline‐HPV seropositive males was greater than those who were baseline seronegative, and that there was an inconclusive but positive association between HPV antibody titer and incident HPV16 detection [20]. This is in line with our current findings; since naturally developed antibody titers do not protect against subsequent HPV infection in males, the individual remains at risk, which may thus translate to continued risk for their female partner.

On the contrary, while statistical precision was an issue, the odds of genital HPV detection in males “at risk” of HPV infection (most constrained analytical framework) was substantially lower, albeit non‐significantly, among those with high female partner baseline serostatus relative to low. This may suggest that while naturally developed antibodies may not have direct protective effects for males, they may benefit from partial protection from having sex with females with high circulating antibody levels, which could potentially mediate transmission. Numerous studies at the individual level found some protection from naturally developed antibodies among females, while others found none, as noted by the meta‐analysis [20]. Overall, naturally acquired HPV16 seropositivity was suggested to provide modest protection against incident HPV16 detection in females (pooled risk = 0.70, 95% CI: 0.61–0.80), but not for other genotypes [20]. Nevertheless, our study did not find definitive protection conferred by female genotype‐specific HPV seroreactivity. Potential associations between female baseline HPV16 serostatus and male HPV detection could have been masked upon considering genotype‐specific detections of the 14 genotypes together; however, our results are in line with previous research in that protective effects of baseline HPV seropositivity in females and subsequent detection were not substantial, nor found for other HPV genotypes [13, 14, 15, 20]. In future studies, it would be worthwhile to determine if these antibodies subsequently impact HPV persistence and clearance in sexual partners.

The nonsignificant positive association between male partner serostatus and female incident HPV detection, and nonsignificant negative association between female partner serostatus and male HPV detection require cautious interpretation. Such results may have been due to chance, given the potential insufficiency in power from the smaller sample of participant‐HPV units “at risk” (i.e., moderately and most constrained analytical frameworks). Though restricted analyses were adjusted for sexual behavior characteristics, estimates may have been confounded by residual and/or other unmeasured variables, like the presence of other sexually transmitted infections. Alternatively, some studies have described pronounced levels of immune responses elicited by an infection at mucosal (vagina) sites compared to keratinized (penile) anatomic sites, suggesting stronger serological responses at the former which may play a factor in the differential associations by sex [30, 31]. However, further research is needed to explore possible differences in immune responses that could explain these discordances.

Nevertheless, this exploration of HPV infection risk and transmission by sex was made possible by the HITCH study's unique couple‐based design, along with the use of analytical frameworks with varying stringency criteria to evaluate the consistency of results and robustness of our findings. Despite these strengths, a few limitations must be acknowledged, some of which have been discussed previously [5, 32, 33]. These include the potential contamination of true HPV infections due to recent sexual activity, possibility of false‐positive and false‐negative HPV detections, and inability to ensure that HPV transmission was between current HITCH partners rather than concurrent or previous ones. In addition, the timing of an incident detection may not have been exact due to the approximate 4‐month interval between study visits. Since males only attended two study visits due to monetary constraints, we were limited in our ability to explore both cumulative and incident HPV detections among male‐HPV units. Although using data from the first follow‐up visit as proxies for baseline could introduce error, the proximity of the two visits (~4 months) permits the assumption that infection status would be the same. Moreover, the percentage of missing baseline serological and HPV DNA positivity data was < 3% for both females and males. Therefore, any potential limitations were negligible, as confirmed by the robustness of our findings in the sensitivity analyses.

Of note, the reliability of self‐collected samples from females was not validated against the nurse‐collected samples from males. However, the participating females were provided with detailed self‐sampling instructions, and self‐collected vaginal samples have been shown to be comparable to provider‐collected samples when using PCR assays [34]. While HPV L1 antibodies tend to be genotype‐specific, there may be a degree of antibody cross‐reactivity for more closely related genotypes, like HPVs 6 and 11 [35]; however, cross‐reactive antibody titers are typically lower than genotype‐specific antibody titers [36]. Despite this, cross‐reactive antibodies potentially provide some cross‐protection, contributing to the antibody‐mediated response against a certain genotype and playing a relevant role in seroreactivity [7].

Overall, we found that one's genotype‐specific HPV serostatus was not associated with their sexual partner's risk of cumulative and incident HPV detections in our longitudinal cohort of unvaccinated, young, recently formed, sexually active, heterosexual couples. Such results align with the existing understanding that HPV virions from natural infection, which are relative immune evaders and typically confined to epithelial cells, elicit only modest immune responses that may not be sufficient for robust protection [37]. In contrast, the primary preventive measure that involves HPV virus‐like proteins administered through targeted and optimized vaccination provides a highly immunogenic trigger; completion of the full HPV vaccination schedule produces serum neutralizing antibody titers 10‐ to 100‐fold higher than those generated by natural infection [38, 39, 40, 41]. Additionally, vaccination induces significant cell‐mediated responses, effectively engaging the two critical axes of the adaptive immune system [37]. Consequently, HPV vaccination not only offers individual protection but also effectively contributes to herd immunity and a lowered incidence of vaccine‐preventable HPV genotypes in the unvaccinated individuals of vaccinated partners [5, 42, 43]. Therefore, our findings showing a lack of association between HPV seroreactivity and risk of HPV infection among unvaccinated sexual partners emphasize the importance of HPV vaccination in providing robust, targeted protection against important HPV genotypes, effectively reducing their transmission.

## Author Contributions

E.L.F. and A.N.B. led the HITCH study. P.‐P.T. and F.C. acted as co‐investigators. F.C. was responsible for HPV DNA testing. T.W. was responsible for HPV multiplex serology testing. M.D.W., M.E.‐Z., and E.L.F. conceptualized the protocol for the serology testing component of the HITCH study. K.N., M.E.‐Z., A.N.B., and M.D.W. accessed and managed the data. K.N. conducted the statistical analyses and drafted the manuscript under the supervision and guidance of M.E.‐Z. and E.L.F. All authors had access to and interpreted results and critically reviewed the manuscript.

## Ethics Statement

Ethical approval was obtained from the committees at McGill University, Concordia University, and the Centre Hospitalier de l'Université de Montréal, and is annually renewed at McGill University (Institutional Review Board Study Number A09‐M77‐04A).

## Consent

All participants provided written informed consent.

## Conflicts of Interest

Eduardo L. Franco has served as an occasional advisor to companies involved with human papillomavirus (HPV) diagnostics (Roche, BD, Qiagen, Gen‐Probe) and HPV vaccines (Merck, GSK). His institution has received unconditional grants from Merck in aid of investigator‐initiated studies. Mariam El‐Zein and Eduardo L. Franco hold a patent related to the discovery “DNA methylation markers for early detection of cervical cancer,” registered at the Office of Innovation and Partnerships, McGill University, Montreal, Quebec, Canada (October 2018). François Coutlée has received grants or free reagents through his institution from Merck Sharp and Dohme, Becton Dickinson and Roche, as well as honoraria from Merck and Roche for lectures on HPV. Tim Waterboer serves on advisory boards for MSD (Merck) Sharp & Dohme. The remaining authors declare no conflicts of interest. None of the work presented in this manuscript was paid for or influenced by any of the aforementioned companies.

## Supporting information

Supporting information.

## Data Availability

HITCH participant consent forms stated that data would be published in aggregate form and individual‐level data would only be available to study investigators upon request. To access data, please contact Eduardo Franco at eduardo.franco@mcgill.ca. The protocol for the HITCH cohort study has been published [21].
