# Supplementary material for: Naturally Acquired Genotype‐Specific HPV Seroreactivity and Subsequent HPV Detection Among Heterosexual Partners
Source: J Med Virol. 2025 Jan 15;97(1):e70163. doi: 10.1002/jmv.70163 (PMC11733845; doi:10.1002/jmv.70163)
Supplement: Supplementary file 1 — Supporting information. [file JMV-97-e70163-s001.docx]

**Table S1. Forward stepwise logistic regression^a^ coefficients for couple baseline sexual behaviour characteristics, used to construct a propensity score to detect any HPV positivity in females at baseline (n=390).**

| **Retained variables^b^** | **Coefficient (95% CI)** | **Standard error** |
| --- | --- | --- |
| Female participant’s number of lifetime vaginal sex partners^c^ | 0.14 (0.09, 0.19) | 0.03 |
| Male participant’s number of lifetime vaginal sex partners^c^ | 0.06 (0.03, 0.09) | 0.02 |
| Female participant’s weekly frequency of sexual activity^c^ | 0.05 (-0.01, 0.10) | 0.03 |
| Female participant has concurrent partners | 0.35 (-0.17, 0.88) | 0.27 |
| Constant | -2.02 (-2.55, -1.49) | 0.27 |

CI, confidence interval

^a^ Sexual behaviour characteristics were in the logistic regression model if the p-value was <0.2, using the Wald test.

^b^ Sexual behaviour characteristics for both female and male participants in a couple included: marital status, number of lifetime vaginal sex partners, number of lifetime sex partners (anal, oral, and/or vaginal), time between first sex and 1^st^ visit, condom use, concurrent partners (yes/no), weekly frequency of vaginal sexual activity, and weekly frequency of sexual activity.

^c^ Missing values for each variable were imputed using the mean value of a given variable.

**Table S2. Forward stepwise logistic regression^a^ coefficients for couple baseline sexual behaviour characteristics, used to construct a propensity score to detect any HPV positivity in males at baseline (n=389).**

| **Retained variables^b^** | **Coefficient (95% CI)** | **Standard error** |
| --- | --- | --- |
| Male participant’s number of lifetime vaginal sex partners^c^ | 0.11 (0.07, 0.15) | 0.02 |
| Female participant’s number of lifetime vaginal sex partners^c^ | 0.08 (0.03, 0.13) | 0.02 |
| Condom use (1-25% of the time), according to female participant^c^ | 0.68 (0.19, 1.18) | 0.25 |
| Condom use (26-75% of the time), according to male participant^c^ | 0.60 (-0.04, 1.23) | 0.32 |
| Constant | -2.01 (-2.47, -1.54) | 0.24 |

CI, confidence interval

^a^ Sexual behaviour characteristics were in the logistic regression model if the p-value was <0.2, using the Wald test.

^b^ Sexual behaviour characteristics for both female and male participants in a couple included: number of lifetime vaginal sex partners, number of lifetime sex partners (anal, oral, and/or vaginal), time between first sex and 1^st^ visit, condom use, weekly frequency of vaginal sexual activity, and weekly frequency of sexual activity.

^c^ Missing values for each variable were imputed using the mean value. For categorical variables, an arbitrary value outside of the categories was used to signify a missing value.


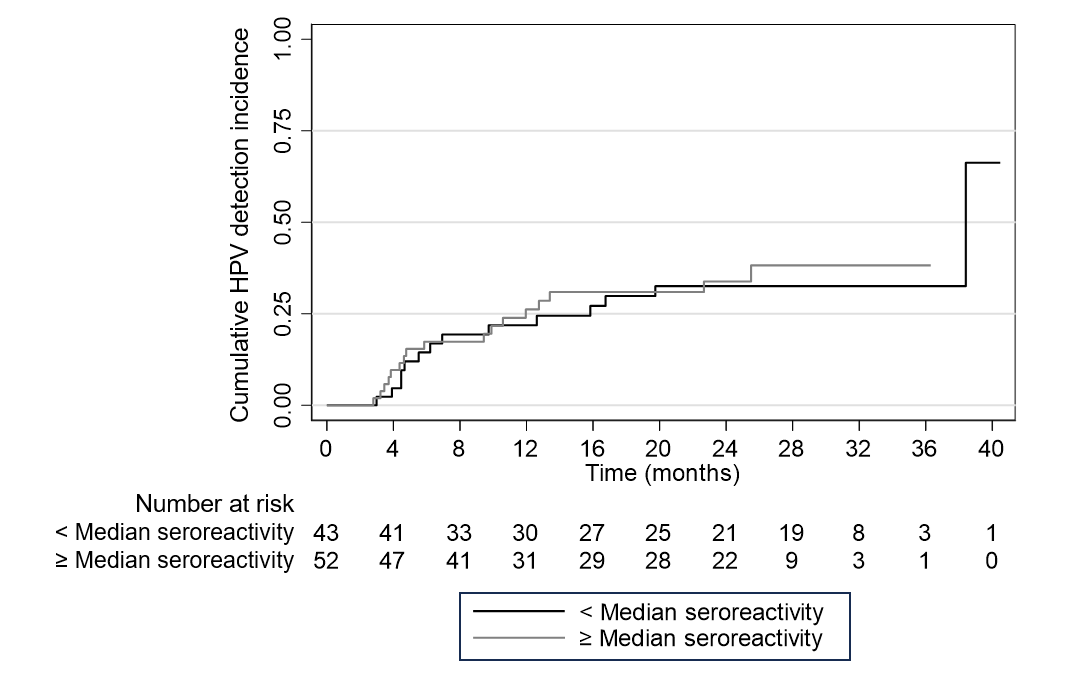


**Figure S1.** **Kaplan-Meier failure curves of incident female genotype-specific HPV detections by male partner serostatus defined by the median antibody titer, among all female-HPV units who are HPV-negative at baseline with an HPV-positive male partner.**

For the most constrained analytical framework, 95 female-HPV units initially tested HPV-negative at baseline but were susceptible to acquiring an infection from an HPV-positive male. The hazard function for female-HPV units was plotted according to the corresponding male genotype-specific HPV serostatus, defined as below or above the male median baseline antibody titer for the corresponding HPV genotype. Female-HPV units with low and high male genotype-specific serostatuses are in dark and light grey, respectively.
